# Quantitatively Partitioning Microbial Genomic Traits among Taxonomic Ranks across the Microbial Tree of Life

**DOI:** 10.1128/mSphere.00446-19

**Published:** 2019-08-28

**Authors:** Taylor M. Royalty, Andrew D. Steen

**Affiliations:** a Department of Earth and Planetary Sciences, University of Tennessee, Knoxville, Tennessee, USA; b Department of Microbiology, University of Tennessee, Knoxville, Tennessee, USA; U.S. Department of Energy Joint Genome Institute

**Keywords:** metagenomes, taxonomy, uncultured

## Abstract

Widely used microbial taxonomies, such as the NCBI taxonomy, are based on a combination of sequence homology among conserved genes and historically accepted taxonomies, which were developed based on observable traits such as morphology and physiology. A recently proposed alternative taxonomy database, the Genome Taxonomy Database (GTDB), incorporates only sequence homology of conserved genes and attempts to partition taxonomic ranks such that each rank implies the same amount of evolutionary distance, regardless of its position on the phylogenetic tree. This provides the first opportunity to completely separate taxonomy from traits and therefore to quantify how taxonomic rank corresponds to traits across the microbial tree of life. We quantified the relative abundances of clusters of orthologous group functional categories (COG-FCs) as a proxy for traits within the lineages of 13,735 cultured and uncultured microbial lineages from a custom-curated genome database. On average, 41.4% of the variation in COG-FC relative abundance is explained by taxonomic rank, with domain, phylum, class, order, family, and genus explaining, on average, 3.2%, 14.6%, 4.1%, 9.2%, 4.8%, and 5.5% of the variance, respectively (*P < *0.001 for all). To our knowledge, this is the first work to quantify the variance in metabolic potential contributed by individual taxonomic ranks. A qualitative comparison between the COG-FC relative abundances and genus-level phylogenies, generated from published concatenated protein sequence alignments, further supports the idea that metabolic potential is taxonomically coherent at higher taxonomic ranks. The quantitative analyses presented here characterize the integral relationship between diversification of microbial lineages and the metabolisms which they host.

**IMPORTANCE** Recently, there has been great progress in defining a complete taxonomy of bacteria and archaea, which has been enabled by improvements in DNA sequencing technology and new bioinformatic techniques. A new, algorithmically defined microbial tree of life describes those linkages, relying solely on genetic data, which raises the issue of how microbial traits relate to taxonomy. Here, we adopted cluster of orthologous group functional categories as a scheme to describe the genomic contents of microbes, a method that can be applied to any microbial lineage for which genomes are available. This simple approach allows quantitative comparisons between microbial genomes with different gene compositions from across the microbial tree of life. Our observations demonstrate statistically significant patterns in cluster of orthologous group functional categories at taxonomic levels that span the range from domain to genus.

## Abstract (precis)

Recently, there has been great progress in defining a complete taxonomy of bacteria and archaea, which has been enabled by improvements in DNA sequencing technology and new bioinformatic techniques. A new, algorithmically defined microbial tree of life describes those linkages, relying solely on genetic data, which raises the issue of how microbial traits relate to taxonomy. Here, we adopted cluster of orthologous group functional categories as a scheme to describe the genomic contents of microbes, a method that can be applied to any microbial lineage for which genomes are available. This simple approach allows quantitative comparisons between microbial genomes with different gene compositions from across the microbial tree of life. Our observations demonstrate statistically significant patterns in cluster of orthologous group functional categories at taxonomic levels that span the range from domain to genus.

## INTRODUCTION

The relationship between microbial taxonomy and function is a longstanding problem in microbiology (1–3). Prior to the identification of the 16S rRNA gene as a taxonomic marker, microbial phylogenetic relationships were defined by traits such as morphology, behavior, and metabolic capacity. Inexpensive DNA sequencing has provided the ability to fortify those phenotype-based taxonomies with quantitative determinations of differences between marker genes, but canonical taxonomies such as the NCBI taxonomy continue to “reflect the current consensus in the systematic literature,” which ultimately derives from trait-based taxonomies (4). Recently, Parks et al. (5) formalized the Genome Taxonomy Database (GTDB), a phylogeny in which taxonomic ranks are defined by “relative evolutionary divergence” in order to create taxonomic ranks that have uniform evolutionary meaning across the microbial tree of life (5). This approach removes phenotype or traits entirely from taxonomic assignment, as evolutionary distance is calculated from the alignment of 120 and 122 concatenated, universal proteins found in all bacterial and archaeal lineages, respectively. An investigation of the relationship between traits and phylogeny was possible until the recent publication of a microbial tree of life that is based solely on evolutionary distance. Thus, we ask the following question: to what extent does GTDB phylogeny predict microbial traits?

Comparing phenotypic characteristics of microorganisms across the tree of life is not currently possible, because most organisms and lineages currently lack cultured representatives (6, 7). We therefore used the abundance of different clusters of orthologous groups (COGs) in microbial genomes, a proxy for phenotype which is available for all microorganisms for which genomes are available. The clusters of orthologous groups (COGs) method represents a classification scheme that defines protein domains based on groups of proteins sharing high sequence homology (8). More than ∼5,700 COGs have been identified to date. COGs are placed into 1 of 25 metabolic functional categories (COG-FCs), with each representing a generalized metabolic function (e.g., “lipid transport and metabolism” or “chromatin structure and dynamics”). Our analyses quantify the degree to which taxonomic rank (genus through domain) predicts the COG-FC content of genomes and illustrate which lineages are relatively enriched or depleted in specific COG-FCs. These analyses constitute a step toward better understanding of how evolutionary processes influence the distribution of metabolic traits across taxonomy as well as a step toward being able to probabilistically predict the metabolic or functional similarity of microbes given their taxonomic classification.

## RESULTS

The genomes analyzed in this work were compiled from a variety of different sources, including RefSeq v92, the JGI Integrated Microbial Genomes and Microbiomes (IMG/M) database, and GenBank, in order to include genomes created using diverse sequencing and assembly techniques. The integration of the RefSeq v92, JGI IMG/M, and GenBank databases resulted in a total of 119,852 genomes within the custom-curated database. Raw data, GTDB taxonomy, and associated accession numbers are provided in Data Set S1 (available at https://zenodo.org/record/3361565). Among these genomes, we included only those that satisfied a set of criteria designed to ensure that each genus contained enough genomes to allow statistically robust analysis (see Materials and Methods). This resulted in a set of 13,735 lineages, representing 22 bacterial phyla and 4 archaeal phyla, 67% of which have been grown in culture (Table 1).

**TABLE 1:** A summary of the custom-curated genome database used in this work

Unique domain	Unique phyla	No. of unique classes	No. of unique orders	No. of unique families	No. of unique genera	No. of unique lineages	No. of cultured lineages	No. of uncultured lineages
*Bacteria*	*Actinobacteriota*	3	9	22	50	2,286	2,115	171
	*Bacteroidota*	3	7	19	50	1,606	741	865
	*Campylobacterota*	1	1	6	8	270	203	67
	*Cyanobacteria*	2	3	4	7	119	84	35
	*Deinococcota*	1	1	2	2	44	44	0
	*Desulfobacterota*	2	2	2	4	43	23	20
	*Elusimicrobiota*	1	1	1	1	22	0	22
	*Fibrobacterota*	1	1	1	1	34	22	12
	*Firmicutes*	3	10	23	48	1,543	1,356	187
	*Firmicutes* A	2	7	10	31	600	304	296
	*Firmicutes* B	1	1	1	1	22	11	11
	*Firmicutes* C	1	2	3	4	53	32	21
	*Fusobacteriota*	1	1	2	2	40	40	0
	*Marinisomatota*	1	1	1	1	10	0	10
	*Nitrospirota*	2	2	2	2	30	6	24
	*Nitrospirota* A	1	1	1	1	14	2	12
	*Patescibacteria*	6	16	26	36	707	0	707
	*Proteobacteria*	3	25	59	163	5,589	3,952	1,637
	*Spirochaetota*	3	4	4	6	153	89	64
	*Synergistota*	1	1	1	1	19	2	17
	*Thermotogota*	1	1	2	2	23	15	8
	*Verrucomicrobiota*	2	4	5	7	84	16	68

*Archaea*	*Crenarchaeota*	1	1	1	2	68	7	61
	*Euryarchaeota*	2	2	2	2	45	26	19
	*Halobacterota*	4	5	7	9	164	97	67
	*Thermoplasmatota*	1	1	2	9	147	0	147

Total	26	50	110	209	450	13,735	9,187	4,548

**FIG 1:**
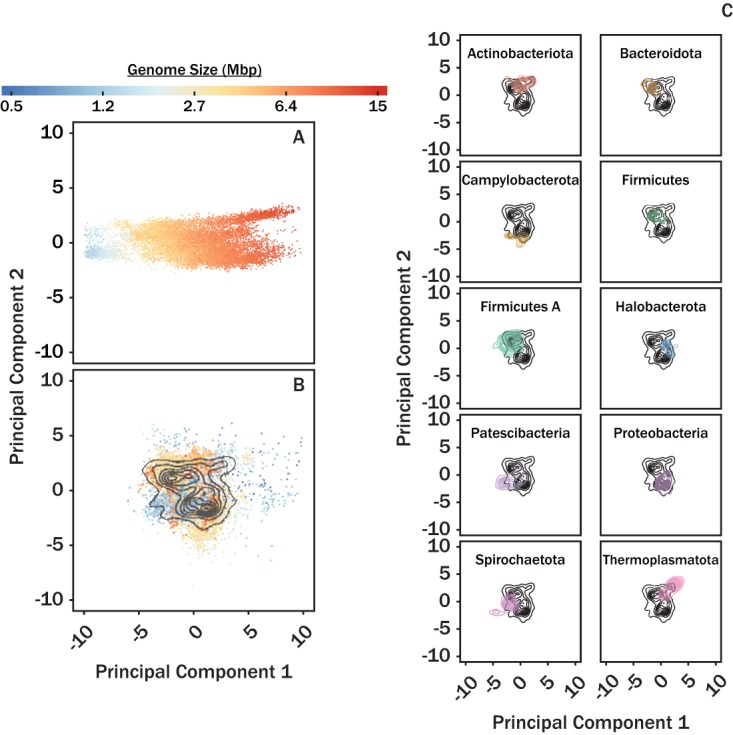
PCA plots of COG-FC abundances (A) and relative abundances (B and C). Individual data points are colored by genome size in panels A and B. The data presented in panel A were not normalized by genome size, while the data in panels B and C were normalized by genome size. Black contours on panels B and C correspond to density plots for all genomes shown in panel B. Colored contours in panel C correspond to the respective lineage labels. For panel A, PC1 explained 71% and PC2 explained 7.0% of the variance. For panels B and C, PC1 explained 21% and PC2 explained 16% of the variance. Panel C corresponds to only the top 10 most abundant phyla analyzed as described for Table 1, while the remaining contours are shown in Fig. S3.

Most predicted open reading frames for most lineages could be assigned to a COG-FC. Across all phyla, an average of 84.3% ± 7.8% of open reading frames were assigned to a COG-FC (see Fig. S1 in the supplemental material). Genomes of the same phylum tended to group together in an initial principal-component analysis (PCA) of raw COG-FC abundance (data not shown). Since this analysis was based on the absolute abundances of COG-FCs in genomes, rather than on the relative abundances, we hypothesized that the relationship between COG-FC abundance and phylum was largely a consequence of genome size, which is phylogenetically conserved (9). Consistent with this possibility, position on PC 1 correlated closely with genome size (*R*^2^ = 0.88; Fig. 1A). We therefore normalized each COG-FC abundance value, for each genome, to a prediction of COG-FC abundance as a function of genome size derived from a generalized additive model (GAM) (Fig. S2; summary statistics are listed in Table S1 in the supplemental material). The data associated with each GAM model were statistically significant (*P < *0.001), and all but five COG-FCs had deviance-explained values (analogous to adjusted *R*^2^ values) of more than 50%. We interpret analyses of these genome size-normalized data sets as reflecting the enrichment or depletion of COG-FC abundance, relative to that expected for a given genome size, and thus, the data are defined as COG-FC relative abundances. PCA of these COG-FC relative abundances showed that species-level lineages still tended to group by phylum, even though the interphylum gradients in genome size were no longer apparent (Fig. 1B and C). Note that attempts were made to normalize by genome size alone; however, these attempts failed to properly remove the influence of genome size. We hypothesize that this was due to the nonlinear response in COG-FC abundances as a function of genome size.

10.1128/mSphere.00446-19.1 FIG S1 Violin plots showing the distribution for the ratio of total COG-FC annotations in a genome to the total number of open reading frames for each phylum. Download FIG S1, PDF file, 0.6 MB. Copyright © 2019 Royalty and Steen. 2019 Royalty and Steen This content is distributed under the terms of the Creative Commons Attribution 4.0 International license.

10.1128/mSphere.00446-19.2 FIG S2 GAM regressions modeling COG-FC normalized abundance (standardized) as a function of genome size. Solid red lines correspond to mean fit. Upper and lower red dashed lines correspond to 95th percentile confidence intervals. Download FIG S2, JPG file, 2.3 MB. Copyright © 2019 Royalty and Steen. 2019 Royalty and Steen This content is distributed under the terms of the Creative Commons Attribution 4.0 International license.

10.1128/mSphere.00446-19.3 FIG S3 Contour plots similar to those shown in Fig. 1C. Lineages shown are for those listed in Table 1 that are not shown in Fig. 1C. Download FIG S3, PDF file, 0.9 MB. Copyright © 2019 Royalty and Steen. 2019 Royalty and Steen This content is distributed under the terms of the Creative Commons Attribution 4.0 International license.

10.1128/mSphere.00446-19.4 TABLE S1 Fit statistics for GAM regressions modeling COG-FC abundance as a function of genome size. Download Table S1, XLSX file, 0.01 MB. Copyright © 2019 Royalty and Steen. 2019 Royalty and Steen This content is distributed under the terms of the Creative Commons Attribution 4.0 International license.

To quantify the degree to which taxonomic rank explains the distribution of COG-FC relative abundances among individual genomes, we performed permutation multivariate analysis of variance (PERMANOVA) using the taxonomic ranks of domain, phylum, class, order, family, and genus, as well as culture status (cultured versus uncultured lineage). The rank of species was excluded from the analysis as every lineage was unique, and thus, species would explain 100% of the data. Every rank significantly influenced the distribution of COG-FC relative abundances (*P < *0.001), but the fractions of variance that the various ranks explained differed substantially: phylum explained the most variance (14.6%), followed by order (9.2%), genus (5.5%), family (4.8%), and class (4.1%). Domain explained only 3.1% of the variances in COG-FC relative abundances, the least of any taxonomic rank. Culture status was a significant correlate of COG-FC abundance (*P < *0.001) but had virtually no explanatory power, accounting for only <0.001% of the variance. This observation is consistent with the idea that no particular COG-FC relative abundance level was systematically higher or lower in uncultured microbes relative to cultured microbes.

The variability in COG-FC relative abundances across different phyla was explored in addition to mean COG-FC composition for individual phyla (see Fig. 3). The evolutionary distance in COG-FC content was measured for all lineages in respect to the phylum COG-FC centroid (see Fig. 3A). The variations in calculated distances for all lineages within a given phylum were compared across the entire phylum (see Fig. 3A). Among all phyla, the *Crenarchaeota* differed the most from the phylum centroid, indicating the largest amount of genomic variation in terms of COG-FC content, followed by *Patescibacteria* and *Cyanobacterota*. The least variable phyla were the *Synergistota*, *Marinisomatota*, and *Fibrobacterota* phyla, respectively (see Fig. 3A). We explored the possibility that the amount of variance in lineages from the phylum centroid was a function of the number of lineages in the phylum. In other words, did the COG-FC content of some genomes seem less variable simply because they had been undersampled? A plot of the average distance of lineages from their phylum’s centroid (i.e., the center of the mass of all genomes in the trait space) versus the number of lineages in the phylum reveals that increased sampling caused an apparent increase in the variability of traits within a phylum. This increase in variability across the phylum began to resemble an asymptotic line after approximately 100 genomes were sampled (see Fig. 3B). We modeled the data using both a saturating model (equation 1) and a linear model to test this observation. The saturating model described the relationship substantially better than a linear regression, as determined by the Akaike information criterion (AIC; ΔAIC = 10.5). Coefficient A of the saturating model, which represents the value of the asymptote, was estimated to be 0.75 ± 0.15 (*P* < 0.001). Coefficient B, which represents how quickly the function approaches the asymptote, was 0.43 ± 0.30 (*P* = 0.17). Coefficient C, representing an offset employed to address the fact that all the log-transformed distances have negative values, was −1.63 ± 0.14 (*P* < 0.001). This means that observing approximately 100 lineages in a phylum is sufficient to assess the variance in trait space representing half of all potential variance for that phylum (0.13). Note that this represents the effect of incorporation of the shift parameter, coefficient C.

We sought a qualitative determination of how the distribution of COG-FC relative abundances related to phylogeny. To achieve this, we quantified the average COG-FC relative abundances for all COG-FCs in each genus These values were then visualized on a genus-level phylogenetic tree (see Fig. 4) utilizing concatenated ribosomal protein sequences published previously by Parks et al. (5). Data underlying the genus-level phylogenetic tree (see Fig. 4) are presented in Table S2. Several notable features appear in COG-FC relative abundances at the phylum level. For example, among the four archaeal phyla represented here, *Thermoplasmatota* appears unique, with high COG-FC relative abundances in cell motility and depletion in every other category. In general, the COG-FC content of bacterial lineages appeared more variable than that of the archaeal lineages at all taxonomic resolutions. The clade consisting of *Bacteroidota*, *Spirochaetota*, and *Verrucomicrobiota* was notably depleted in the less variable COG-FCs, including energy production and conversion, amino acid transport and metabolism, and carbohydrate transport and metabolism, among others. Another prominent feature is the nearly ubiquitous elevation in COG-FC relative abundances of cell motility; secondary metabolite biosynthesis, transport, and catabolism; lipid transport and metabolism; and intracellular trafficking, secretion, and vesicular transport COGs in *Proteobacteria*. A notable dichotomy in the COG-FC relative abundances of RNA processing and modification within the *Proteobacteria* mirrors the division of the two largest clades within the *Proteobacteria*. Overall, the relative abundance data appear qualitatively consistent with phylogenetic relationships, albeit they occur on different taxonomic levels.

10.1128/mSphere.00446-19.5rev CORRECTED TABLE S2 Individual rows correspond to individual genus-level lineages (excluding the top row, which consists of column headers). Columns 1, 2, 3, 4, and 5 correspond to domain, phylum, order, family, and genus, respectively. Columns 6 through 28 correspond to average enrichments for the respective lineages and COG functional categories. Download Table S2, CSV file, 0.2 MB. Copyright © 2019 Royalty and Steen. 2019 Royalty and Steen This content is distributed under the terms of the Creative Commons Attribution 4.0 International license.

10.1128/mSphere.00446-19.5rev CORRECTED TABLE S2 Individual rows correspond to individual genus-level lineages (excluding the top row, which consists of column headers). Columns 1, 2, 3, 4, and 5 correspond to domain, phylum, order, family, and genus, respectively. Columns 6 through 28 correspond to average enrichments for the respective lineages and COG functional categories. Download Table S2, CSV file, 0.2 MB. Copyright © 2019 Royalty and Steen. 2019 Royalty and Steen This content is distributed under the terms of the Creative Commons Attribution 4.0 International license.

**FIG 2:**
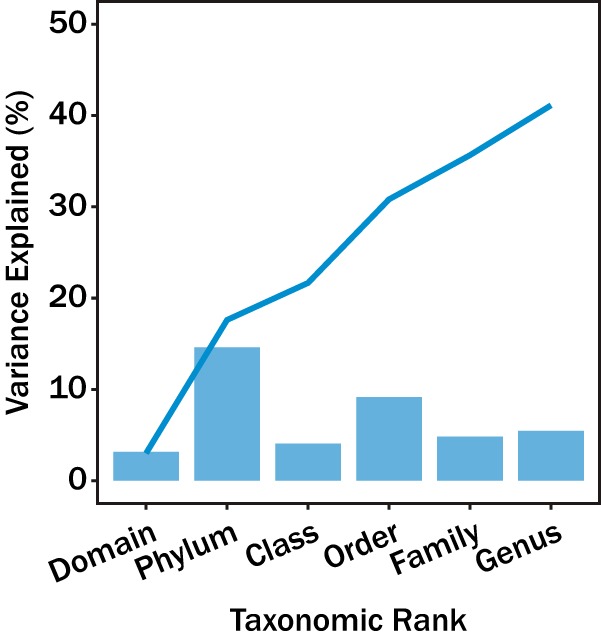
The average variance in COG-FC relative abundance explained by different taxonomic ranks (bars) and the cumulative variance explained by taxonomic ranks (line). All data corresponding to the variance explained by taxonomic ranks were significant (*P < *0.001). The *F*-values for domain, phylum, class, order, family, and genus were 726.0, 128.8, 38.76, 34.4, 11.2, and 5.1, respectively.

The relationship between individual COG-FC relative abundances and taxonomic ranks appeared largely variable (see Fig. 4). For instance, most of the variation in the RNA processing and modification relative abundances occurred at higher taxonomic ranks such as phylum and class whereas most of the variation in the secondary metabolite biosynthesis, transport, and catabolism relative abundances occurred at lower ranks such as order. To quantify this relationship, we applied a variance component model to determine the proportions of the variance explained by different taxonomic ranks (see Fig. 5). Domain and culture status were excluded from this analysis, as determinations of the level of variance explained become imprecise when a factor includes fewer than 5 groups (10). Consistent with the PERMANOVA results (Fig. 2), COG-FC relative abundances were best explained by the taxonomic rank of phylum. In contrast to the PERMANOVA results, the class taxonomic rank appeared to have reasonable explanatory power for a select set of COG-FCs. In general, the overall explanatory power for taxonomic rank appeared to decrease at the lower taxonomic ranks.

Last, to gain a sense of “notable” COG-FCs associated with different phyla, we calculated the mean COG-FC across all lineages in a given phyla and compared these values against the 85th and 15th percentiles for all lineages in our custom-curated database. All COG-FCs whose values were significantly (*P < *0.05; based on a 10^5^ iteration bootstrap analysis) greater or lesser than those calculated for the 85th or 15th percentile, respectively, are shown in Table 2 (see also Table 3). Each archaeal phylum was enriched or depleted in three to nine COG-FCs, whereas most bacterial phyla were enriched or depleted in three to four COG-FCs. A few exceptions arose, including *Fibrobacterota* (depleted in eight COG-FCs), *Nitrospirota* A (enriched in four and depleted in five), and *Proteobacteria* (the only phylum not heavily enriched or depleted in any COG-FCs). Relative abundance data, along with associated GTDB taxonomic assignments used for generating data presented here (see Fig. 4), are provided in Table S2.

**TABLE 2:** Phyla highly enriched (>85th percentile) or depleted (<15th percentile) in COG-FCs^*a*^

Phylum	Enriched COG-FC(s)	Depleted COG-FC(s)
*Actinobacteriota*		4, 6
*Bacteroidota*		11, 18, 22
*Campylobacterota*	4, 6, 10, 15, 19	8, 12
*Cyanobacteria*	19	12
*Deinococcota*		15
*Desulfobacterota*	13	7
*Elusimicrobiota*	3, 10, 15	14, 16
*Fibrobacterota*		7, 11, 12, 13, 16, 18, 20, 22
*Firmicutes*	9, 12, 21	
*Firmicutes* A	9	5, 7, 16, 20
*Firmicutes* B	13, 17, 19	
*Firmicutes* C	19	
*Fusobacteriota*		10, 21
*Marinisomatota*		12, 14
*Nitrospirota*	6, 10, 17	8
*Nitrospirota* A	4, 6, 10, 15	12, 17, 22, 23
*Patescibacteria*		7, 11, 16, 19
*Proteobacteria*		
*Spirochaetota*	4	5, 16, 19
*Synergistota*	3, 4, 11, 16	
*Thermotogota*	3, 4, 8, 10	
*Verrucomicrobiota*	1	10, 12, 14, 21, 23
*Crenarchaeota*	3, 13, 11, 19, 7, 12, 16, 5, 22	9, 10, 14, 15, 17
*Euryarchaeota*	2, 3, 13, 18, 22	5, 7, 10, 14, 15
*Halobacterota*	3, 19, 22	15, 16
*Thermoplasmatota*	1, 2, 7, 13	4, 6, 8, 9, 10, 14, 15, 16

a Data for all reported categories are statistically significant. See Table 3 for COG-FC numbering key.

**TABLE 3:** COG-FC numbering key

COG-FC	COG-FC ID^*a*^
Cytoskeleton	1
RNA processing and modification	2
Chromatin structure and dynamics	3
Cell motility	4
Secondary metabolite biosynthesis, transport, and catabolism	5
Intracellular trafficking, secretion, and vesicular transport	6
Lipid transport and metabolism	7
Carbohydrate transport and metabolism	8
Defense mechanism	9
Signal transduction mechanisms	10
Amino acid transport and metabolism	11
Transcription	12
Energy production and conversion	13
Replication, recombination, and repair	14
Cell wall/membrane/envelope biogenesis	15
Inorganic ion transport and metabolism	16
Cell cycle control, cell division, and chromosome partitioning	17
Function unknown	18
Coenzyme transport and metabolism	19
Posttranslational modification, protein turnover, and chaperone	20
Nucleotide transport and metabolism	21
General function prediction only	22
Translation ribosomal structure and biogenesis	23

a ID, identifier.

## DISCUSSION

We observed that the abundance of COG-FCs within individual lineages tentatively grouped according to phylum after variable reduction was performed via PCA (data not shown). Furthermore, PCA scores along PC1 correlated strongly with genome size (*R*^2^ = 0.88; Fig. 1A). The conserved nature of genome size across phylogeny (9) implies that phylogenic groupings may be an artifact of genome size. Thus, normalization of COG-FC abundances by genome size was performed to properly characterize the relationship between COG-FC and phylogeny. We performed the normalization using the slope from a GAM regression which modeled COG-FC abundance as a function of genome size. The COG-FC normalization removed the influence of genome size (*R*^2^ = 0; Fig. 1B) while retaining phylogenic groupings (Fig. 1C; see also Fig. S3 in the supplemental material).

**FIG 3:**
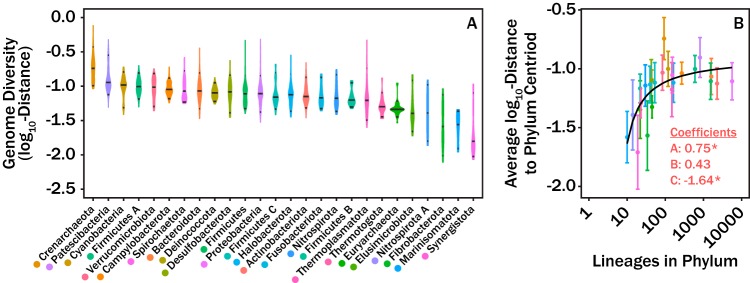
Violin plots showing the distribution of distances (log_10_ transformed) of lineages from their respective phylum centroids (A) and the average of the distances (log_10_ transformed) separating individual lineages from their respective phylum centroids (B). Coefficients in panel B correspond to fit parameters from equation 1. Error bars in panel B correspond to one standard deviation. The asterisks (*) denote significance as defined in the text. We note three outliers: the *Crenarchaeota* are characterized by unusually high diversity of COG-FC distributions, and the *Synergistota* and *Fibrobacterota* are characterized by unusually low diversity of COG-FC distributions.

The PERMANOVA (Fig. 2) and analysis of diversity of genomic composition within phyla (Fig. 3) showed that the microbial lineages exhibited characteristic relative abundances of COG-FC and that the extent of variation varied among taxonomic ranks. Among all the taxonomic ranks, phylum was the most powerful predictor of COG-FC relative abundances, which is consistent with observations that phylum can be informative of microbial function (see, e.g., references 11–13). Lower taxonomic ranks such as genus and family had approximately half the explanatory power shown by the phylum taxonomic rank. Many studies have focused on metabolic coherence of individual traits and have regularly found traits conserved on the family level (2, 3, 14). The discrepancy between previous observations and our observation likely relates to how we characterized patterns in metabolic potential. These studies characterized trait function based on phenotype observation, protein structures, and pathway components. Such characterizations are effective metrics for characterizing finer units of taxonomy, such as genus, but do not scale to coarser units of taxonomy, such as phylum. In contrast, COG-FCs provide a coarse metabolic description which scales with the coarser units of taxonomy (35). The trade-off represented by the approach used here is that, by analyzing COG-FCs, we lose information about specific genes or potential metabolic functions but gain the ability to apply a consistent analysis across an entire genome and across the entire microbial tree of life. Thus, the extent to which observed patterns (Fig. 1) reflect phenotypically expressed differences among lineages is unknown. Nonetheless, the statistical robustness of analyses based on the relationship between all taxonomic ranks and COG-FC patterns suggests that evolutionary processes (e.g., horizontal gene transfer, vertical gene transfer, duplications, deletions, etc.) control the preponderance of different COG-FCs across lineages.

The roles that individual evolutionary processes play in influencing COG-FC relative abundances at a given taxonomic rank likely differ. For instance, horizontal gene transfer is more common among the more closely related lineages (15) and thus likely promotes increased levels of similarity at the lower taxonomic ranks. At higher taxonomic ranks, vertical processes may be more important. The asymptote in the mean log_10_ distance from the centroid as a function of lineages in a phylum suggests that identifying more lineages for larger numbers of poorly represented lineages should expand the diversity of COG-FCs that are found, whereas phyla that were adequately sampled (with at least ∼1,000 lineages) exhibited comparable levels of variability in COG-FC distributions (Fig. 3B). Since many more than 1,000 distinct lineages of each phylum are likely to exist (16), we propose that the taxonomic rank of phylum implies a fairly consistent degree of diversity in COG-FC distribution. To the extent that phenotype matches genotype at the level of COG-FC distributions, we therefore expect that typical phyla exhibit similar levels of phenotypic diversity. A notable exception is the phylum *Crenarchaeota*, whose members were far more diverse than would be expected based on the number of lineages sampled. The *Crenarchaeota*, as defined in the GTDB, collapsed members of several phyla that had been designated separately under previous taxonomies, including lineages that had previously been assigned as *Crenarchaeota*, *Thaumarchaeota*, *Euryarchaeota*, *Verstraetearchaeota*, *Korarchaeota*, and *Bathyarchaeota* (5). It is possible that the relationship between the marker genes used in the GTDB and those in the rest of the genome is unusual for this clade, compared to other phyla, or that the GTDB classification of *Crenarchaeota* is lacking in some other way.

**FIG 4:**
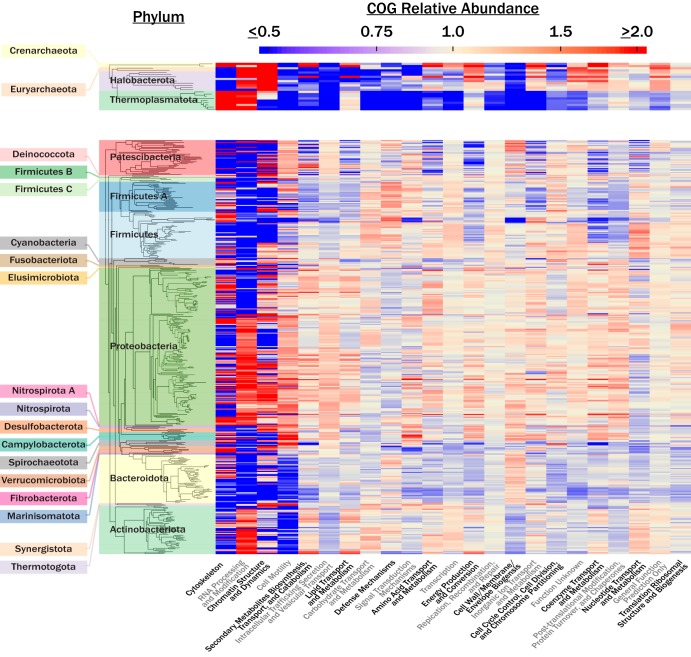
A heat map showing the average COG-FC relative abundances for all archaeal (top) and bacterial (bottom) genera. Categories were arranged from left to right along the *x* axis in order of decreasing total variance in relative abundance across all lineages. Clades were organized along the *y* axis using phylogenetic relatedness based on the concatenated protein sequence alignments reported previously by Parks et al. (5).

**FIG 5:**
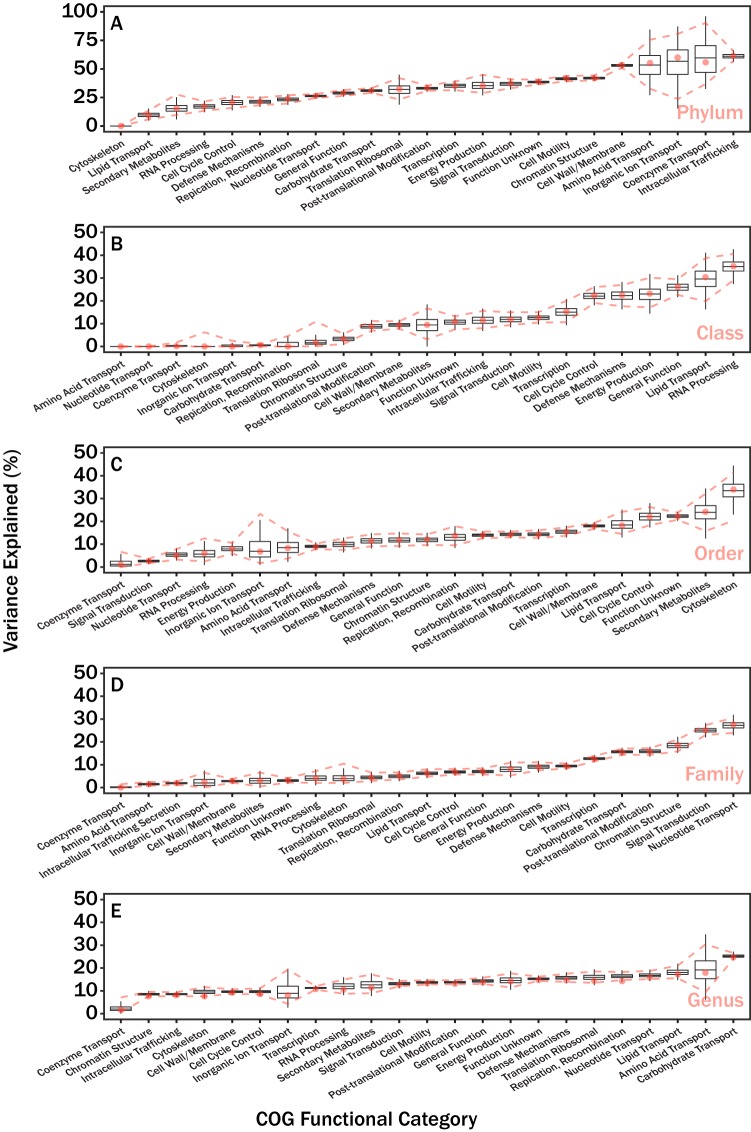
Results from a variance component model. Lineage was used as a nested random effect (intercept) for all COG-FCs. The proportion of variance explained is partitioned by phylum (A), class (B), order (C), family (D), and genus (E). Box plots correspond to the variability in variance explained from the bootstrap analysis, red dashed lines correspond to 95% confidence intervals calculated from the bootstrap analysis, and red circles correspond to the variance explained by analysis of all data in Table 1. Note that the titles for COG-FCs are shortened; the full category names are shown in Fig. 4 and Table 2 (see also Table 3).

Although the genus and family ranks explained relatively little of the variance in COG-FC distribution, examples of consistent colored blocks, indicating that higher or lower relative abundances of specific COG-FCs were conserved across each taxonomic rank in some parts of the phylogenetic tree, are evident in Fig. 4 at every taxonomic resolution. This is explained by the finding of “distantly” related clades (i.e., non-sister clades) occupying similar COG-FC trait space. Our variance component model accounted for the hierarchical nature of taxonomic lineage by partitioning the levels of explanatory power that individual taxonomic ranks had for individual COG-FC relative abundances (Fig. 5). Consistent with Fig. 4, different COG-FCs appeared most controlled at different taxonomic ranks. For instance, the coenzyme transport and metabolism COG-FC was almost entirely explained by the taxonomic rank of phylum. This observation is consistent with previous assessments suggesting that enzyme cofactors are deeply conserved at the phylum level (17, 18). Similarly, the carbohydrate transport and metabolism COG-FC was best explained the taxonomic ranks genus and family, which is consistent with previous observations revealing that large amounts of variability exist for hydrolase traits at the lower taxonomic ranks (1–3). Ultimately, the variability in explanatory power for COG-FCs represented by the different taxonomic ranks supports the notion that evolutionary processes operate on microbial metabolisms at different timescales depending on which component of the metabolism is in question.

The coherence in metabolic potential at higher taxonomic ranks may help explain the distribution of microbial clades across ecological niches. Analyses of habitat associations (1, 9, 19) found phylum-level patterns in lineages occupying niches, which supports the idea that there is a relationship between higher taxonomic ranks, metabolism, and niche. Our analysis provided quantitative evidence supporting this idea by demonstrating coherence in metabolic potential with broad-scale patterns in genomic data (Fig. 1; see also Fig. 5). The following question remains: how well do the observed COG-FC relative abundances reflect expressed functional traits (i.e., phenotypes) across these lineages? It is difficult to address this question systematically, but some of the relative abundances and depletions reported here appear consistent with known physiologies of clades. For instance, *Rickettsiales* were depleted in nucleotide metabolism and transport, consistent with a previously observed lack of a metabolic pathway for purine synthesis among five example *Rickettsiales* species (20). Another example is the depletion in the COG-FCs of energy production and conversion, amino acid transport and metabolism, and carbohydrate transport and metabolism within the *Bacteroidetes*, *Spirochaetes*, and *Chlamydiales* clade (NCBI taxonomy to be consistent with citing literature). This clade is known to contain many host-dependent pathogens and symbionts (21–23) which are often depleted in these COG-FCs (24).

The GTDB classification is the first fully algorithmic and quantitatively self-consistent microbial taxonomy that can be applied across the tree of life (5). By standardizing the meaning of taxonomic ranks, it creates an objective basis on which to compare microbial functionality to phylogeny. The analyses presented here demonstrate that compositional patterns exist for genomic traits which can be explained by different taxonomic ranks. Furthermore, the proportion of variance explained for individual COG-FCs was partitioned as a function of taxonomic ranks. These quantitative relationships allude to the idea that evolutionary processes operate on different timescales for different components of microbial metabolisms and support previous suggestions proposing that a relationship exists between higher taxonomic ranks, metabolism, and ecological niches.

## MATERIALS AND METHODS

### Genome database curation.

All bacterial and archaeal genomes from the RefSeq database v92 (25), all uncultured bacterial and archaeal (UBA) metagenome-assembled genomes (MAGs) reported in Parks et al. (5, 26), all bacterial and archaeal MAGs from the Integrated Microbial Genomes and Microbiomes (IMG/M) database, and all bacterial and archaeal single amplified genomes (SAGs) from IMG/M were curated into a single database. All genomic content within the curated database is referred to as representing a “genome(s)” for simplicity. Genomes were assigned taxonomy consistent with the Genome Taxonomy Database (GTDB) using the GTDB toolkit (GTDB-Tk) v0.2.1 (5). The GTDB-Tk taxonomic assignments were consistent with reference package GTDB r86. Lineages which, due to the absence of a reference lineage, did not receive a genus classification were excluded from analyses. In total, 6.1% of the total number of genomes from the initial database met this condition. Due to bias resulting from the superabundance of strains in specific clades (e.g., Escherichia coli), the lowest taxonomic rank considered during our analysis was species. The COG-FC relative abundances (see below) were averaged for all strains within a given species. An exception was made for lineages which shared a genus classification but lacked a species classification. In this scenario, each genome was treated as an independent lineage. In total, 10.9% of the total number of genomes analyzed (i.e., of those that had a genus assignment) met this condition. Last, only genomes belonging to genera with at least 10 unique species in the database were retained. This criterion ensured the availability of enough data to generate meaningful statistics during our PERMANOVA. The final database is summarized in Table 1. The genus-level phylogenetic tree was generated from concatenated protein sequence alignments published in Parks et al. (5).

### COG functional category identification, enumeration, and normalization.

Genes were predicted from individual genomes and translated into protein sequences using Prodigal v.2.6.3 (27). The resulting protein sequences were analyzed for COGs (8). COG position-specific scoring matrices (PSSMs) were downloaded from NCBI’s Conserved Domain Database (27 March 2017 definitions). COG PSSMs were BLASTed against protein sequences with the reverse-position-specific BLAST (RPS-BLAST) algorithm (28). Following a previously reported protocol (28), we used an E value cutoff of 0.01 to assign COGs with RPS-BLAST. The retrieved COGs were assigned to their respective COG functional categories (COG-FCs; 25 in total), and the abundances of the functional categories were tabulated for each genome by the use of cdd2cog (29). The abundance values determined for the individual COG-FCs were normalized by the respective COG-FC standard deviations across all lineages. For the COG-FCs extracellular structures and nuclear structures, the standard deviation was 0. Consequently, data could not be normalized; thus, these two categories were discarded from all analyses.

COG-FC abundances were normalized by their respective regression slopes of COG-FC abundance for a given genome as a function of genome size. COG-FC abundances were modeled as a function of genome size for individual categories using a generalized additive model (GAM) with a smoothing term employed due to the pairwise response to genome size (see Fig. S1 in the supplemental material). We used the gam function from the R package mgcv (30). In some instances, regression fits were visibly skewed by high-leverage data points. High-leverage data were filtered using the influence.gam function in the mgcv package. Data in the 99.5% percentile for influence were excluded in regression analysis but were included in all downstream analyses. All regressions were significant, with *P* values of <0.001.

### Principal-component analysis (PCA).

We performed PCA on the normalized COG-FC abundances and relative abundances. Prior to PCA, assumptions of normality were achieved by performing a boxcox transformation on individual COG-FC abundance and relative abundance distributions with the boxcox function from the R package MASS (31). The resulting distributions were then scaled by the respective COG-FC standard deviations calculated from all genomes. PCA was performed using the princomp function from the R package stats (32).

### Quantifying COG-FC variance explained by taxonomic rank.

We performed permutational multivariate analysis of variance (PERMANOVA) using the adonis function from the R package vegan (33). The taxonomic ranks domain, phylum, class, order, family, and genus as well as culture status were used as test categorical variables for quantifying variances in COG-FC relative abundances explained by the mean taxonomic rank centroids. The test was performed using the test default value, 999 permutations, for each categorical variable. Distances between mean phyla COG-FC relative abundance centroids and the respective genomes within that phylum were calculated by performing an analysis of multivariate homogeneity of dispersions of groups with the betadisper function from the R package vegan (33). The centroid type input was set as the “centroid” (mean). The distance matrix used for both the adonis and betadisper analyses was generated by calculating Euclidean distances for the normalized COG-FC relative abundances.

The mean log_10_ distance from the phylum centroid was determined for each phylum and modeled with the following equation, which represents a hyperbola shifted on the *x* axis to ensure that the mean distance value is zero when *n* = 1:

(1) $${\text{log}}_{10}\left(\text{mean distance}\right)=\frac{A\left[{\text{log}}_{10}\left(n\right)-1\right]}{B+{\text{log}}_{10}\left(n\right)-1}+C$$

where *A*, *B*, and *C* represent fit coefficients and *n* represents the total number of lineages in the given phylum. The Akaike information criterion value was calculated with the fit from equation 1 using the AIC function from the R package stats (32).

A variance component model was performed using the lme function from the R package nlme (34). The proportion of variance explained by the taxonomic ranks, phylum, class, order, family, and genus, was determined for each individual COG-FC. Domain and culture status were not evaluated due to imprecise results generated from factors that only have 2 groups (10). Lineage was treated as a random intercept, where individual taxonomic ranks were nested within one another in a hierarchical manner (R notation: ∼1|phylum/class/order/family/genus). Confidence intervals were determined by performing a 500-iteration bootstrap analysis with the variance component model. During the bootstrap analysis, genomes were randomly sampled with replacement.

### Data availability.

The genomes analyzed for the current study are available in the NCBI RefSeq database (ftp://ftp.ncbi.nlm.nih.gov/refseq/release/). UBA MAGs used for the current study are available under NCBI’s BioProject PRJNA417962 and PRJNA348753. Publicly available JGI IMG/M genomes can be downloaded from the Genome Portal (https://img.jgi.doe.gov/), while the private genomes were acquired from Chad Burdyshaw. Associated genome accession numbers for genomes in the described data sets are available in Data set S1 at https://zenodo.org/record/3361565 (https://doi.org/10.5281/zenodo.3361565).
