# Erratum for Royalty and Steen, “Quantitatively Partitioning Microbial Genomic Traits among Taxonomic Ranks across the Microbial Tree of Life”

**DOI:** 10.1128/mSphere.00637-19

**Published:** 2019-09-11

**Authors:** Taylor M. Royalty, Andrew D. Steen

**Affiliations:** a Department of Earth and Planetary Sciences, University of Tennessee, Knoxville, Tennessee, USA; b Department of Microbiology, University of Tennessee, Knoxville, Tennessee, USA

## ERRATUM

Volume 4, no. 4, e00446-19, 2019, https://doi.org/10.1128/mSphere.00446-19. The original Table S2 file in the supplemental material contained unstandardized data and has now been replaced with a version that contains standardized data, as described in Materials and Methods.

