# Supplementary figures and images for: Quantitatively Partitioning Microbial Genomic Traits among Taxonomic Ranks across the Microbial Tree of Life
Source: mSphere. 2019 Aug 28;4(4):e00446-19. doi: 10.1128/mSphere.00446-19 (PMC6714891; doi:10.1128/mSphere.00446-19)

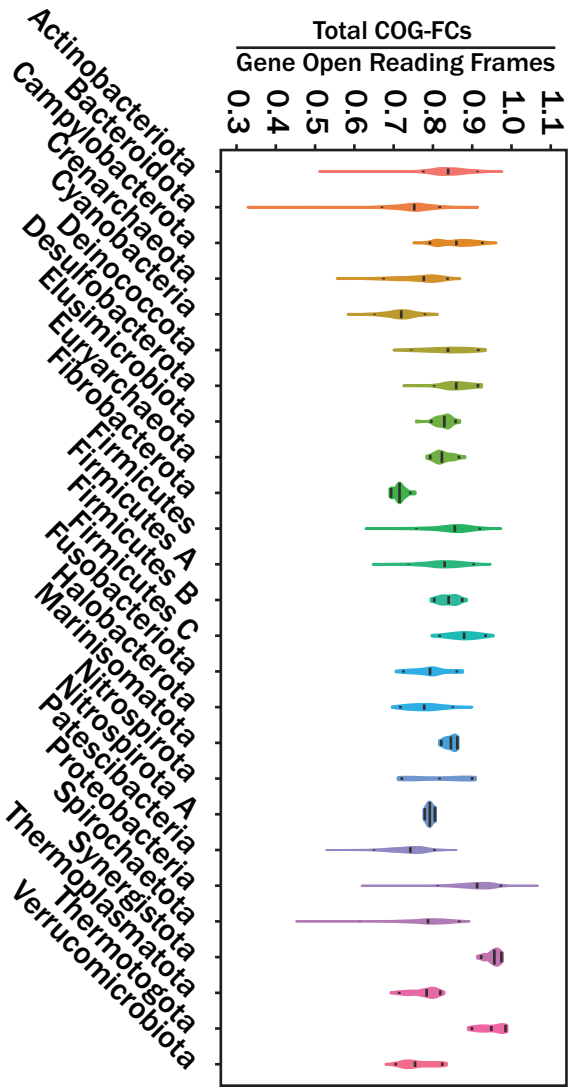

Supplement: FIG S1 [file mSphere.00446-19-sf001.pdf]

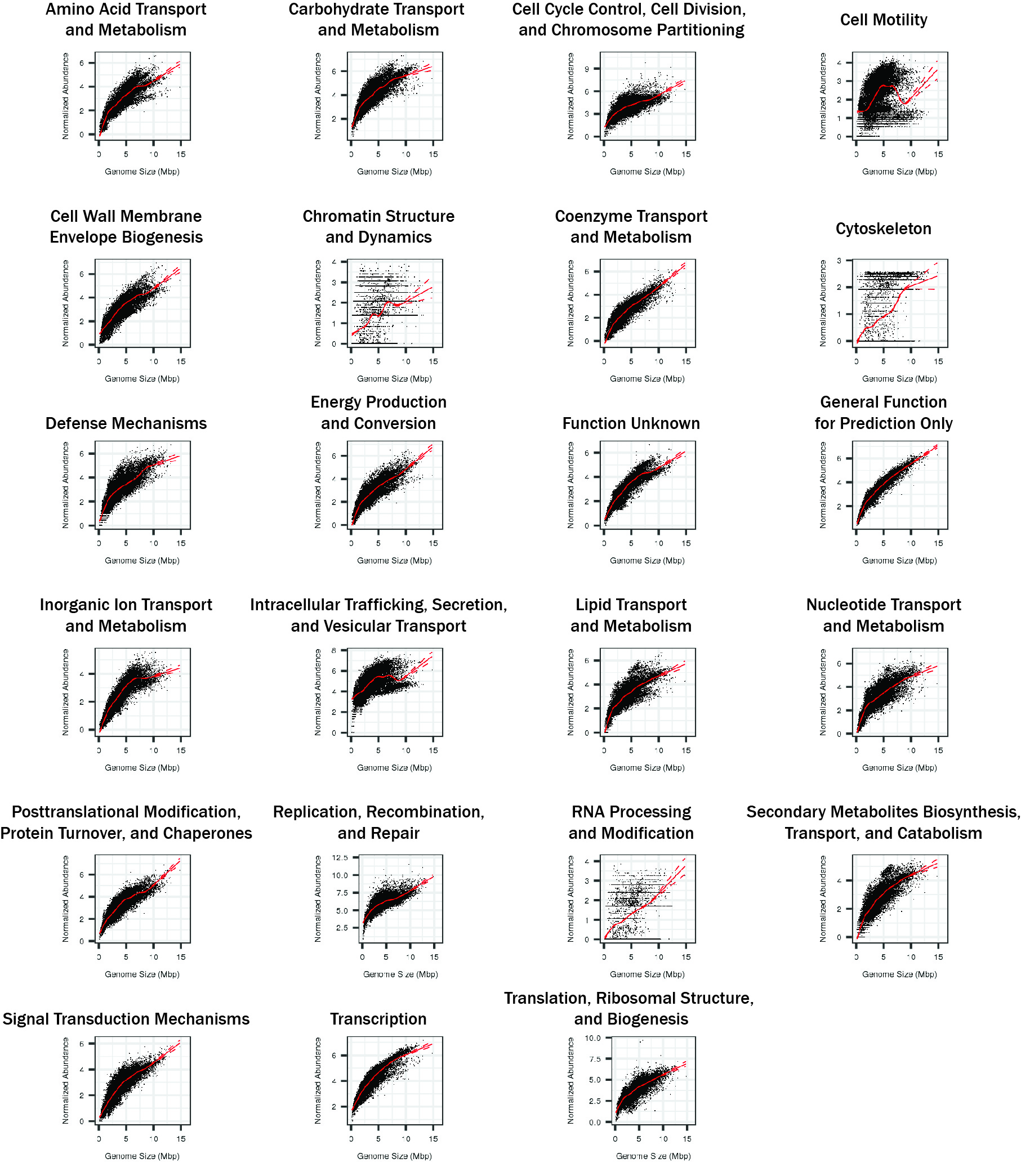

Supplement: FIG S2 [file mSphere.00446-19-sf002.jpg]

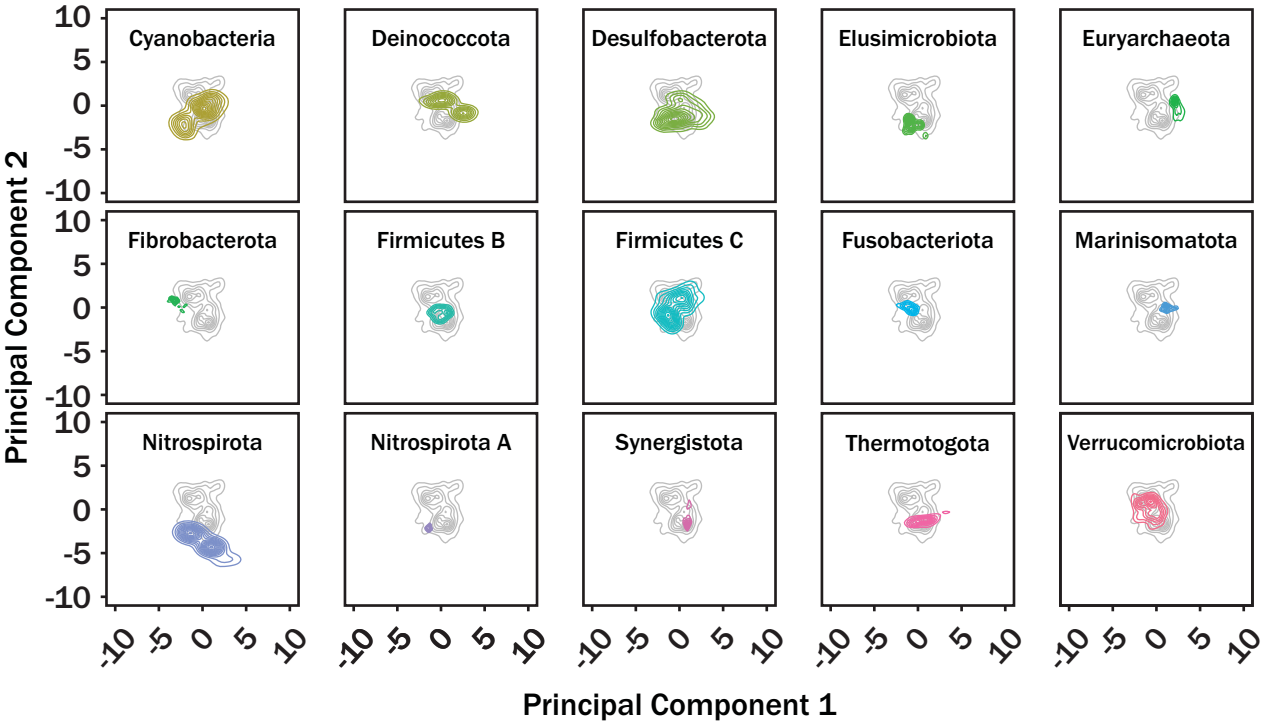

Supplement: FIG S3 [file mSphere.00446-19-sf003.pdf]
